# Supplementary material for: Saccharomyces boulardii (CNCM I-745) improves intestinal damage in sepsis by remodeling intestinal flora structure
Source: Front Cell Infect Microbiol. 2025 Jul 31;15:1602792. doi: 10.3389/fcimb.2025.1602792 (PMC12350329; doi:10.3389/fcimb.2025.1602792)
Supplement: Supplementary file 1 [file DataSheet1.zip › summary/summary/8_Advanced_analyse/2_PICRUSt2_result/COG/Group.BW4_vs_AW4/PICRUSt2_COG_Group.BW4_vs_AW4_diff.pdf]

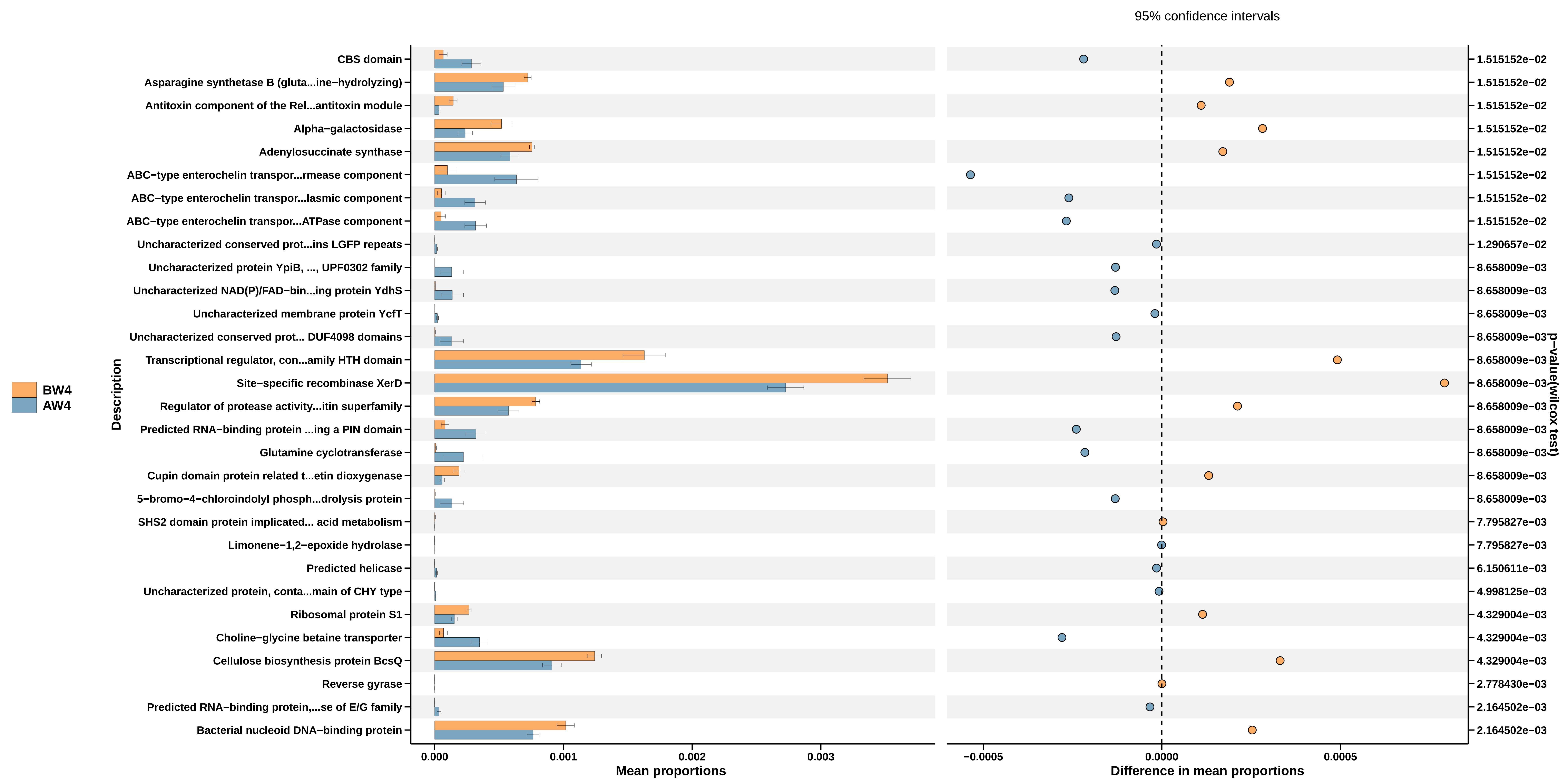

95% confidence intervals
BW4
AW4
Description
CBS domain
Asparagine synthetase B (gluta...ine−hydrolyzing)
Antitoxin component of the Rel...antitoxin module
Alpha−galactosidase
Adenylosuccinate synthase
ABC−type enterochelin transpor...rmease component
ABC−type enterochelin transpor...lasmic component
ABC−type enterochelin transpor...ATPase component
Uncharacterized conserved prot...ins LGFP repeats
Uncharacterized protein YpiB, ..., UPF0302 family
Uncharacterized NAD(P)/FAD−bin...ing protein YdhS
Uncharacterized membrane protein YcfT
Uncharacterized conserved prot... DUF4098 domains
Transcriptional regulator, con...amily HTH domain
Site−specific recombinase XerD
Regulator of protease activity...itin superfamily
Predicted RNA−binding protein ...ing a PIN domain
Glutamine cyclotransferase
Cupin domain protein related t...etin dioxygenase
5−bromo−4−chloroindolyl phosph...drolysis protein
SHS2 domain protein implicated... acid metabolism
Limonene−1,2−epoxide hydrolase
Predicted helicase
Uncharacterized protein, conta...main of CHY type
Ribosomal protein S1
Choline−glycine betaine transporter
Cellulose biosynthesis protein BcsQ
Reverse gyrase
Predicted RNA−binding protein,...se of E/G family
Bacterial nucleoid DNA−binding protein
0.000
0.001
0.002
0.003
Mean proportions
−0.0005
0.0000
0.0005
Difference in mean proportions
p−value(wilcox test)
1.515152e−02
1.515152e−02
1.515152e−02
1.515152e−02
1.515152e−02
1.515152e−02
1.515152e−02
1.515152e−02
1.290657e−02
8.658009e−03
8.658009e−03
8.658009e−03
8.658009e−03
8.658009e−03
8.658009e−03
8.658009e−03
8.658009e−03
8.658009e−03
8.658009e−03
8.658009e−03
7.795827e−03
7.795827e−03
6.150611e−03
4.998125e−03
4.329004e−03
4.329004e−03
4.329004e−03
2.778430e−03
2.164502e−03
2.164502e−03
